# Development and Evaluation of Artificially Attenuated *Corynebacterium pseudotuberculosis*, *Trueperella pyogenes*, and *Staphylococcus aureus* Strains as Live Vaccine Candidates Against Ovine Caseous Lymphadenitis

**DOI:** 10.3390/vetsci13100996

**Published:** 2026-09-22

**Authors:** Bin Wang, Yuchen Wei, Xinxin Qiu, Xindong Bai, Yanqing Jia, Zengqi Yang

**Affiliations:** 1 College of Animal Engineering, Shaanxi A&F Technology University, Yangling 712100, China; wbin0209@126.com (B.W.); wych909@126.com (Y.W.); ylzyqiuxinxin@126.com (X.Q.); 2 The Youth Innovation Team of Shaanxi Universities, Forestry and Animal Husbandry, School of Animal Engineering, Shaanxi Vocational and Technical University of Agriculture, Yangling 712100, China; 3 Shaanxi Engineering & Technology Research Center for Animal Disease Prevention and Control, Yangling 712100, China; 4 Key Laboratory for Efficient Ruminant Breeding Technology of Higher Education Institutions in Shaanxi Province, Yangling 712100, China; 5 Department of Preventive Veterinary Medicine, College of Veterinary Medicine, Northwest A&F University, Yangling 712100, China; baixindong@nwafu.edu.cn

**Keywords:** live attenuated vaccine, polymicrobial infection, serial passage immune response, protective efficacy

## Abstract

Caseous lymphadenitis is a chronic infectious disease causing substantial economic losses to the sheep and goat industry worldwide. The disease is associated with a polymicrobial etiology involving *C. pseudotuberculosis*, *T*. *pyogenes*, and *S*. *aureus*. While inactivated vaccines provide partial protection, live attenuated vaccines offer the advantage of inducing both humoral and cell-mediated immune responses. In this study, we aimed to develop live attenuated vaccine candidates for the three major caseous lymphadenitis-associated pathogens through serial subculturing. Primary strains CpS1-1 (*C. pseudotuberculosis*), TpS1-1 (*T. pyogenes*), and SaS9-1 (*S. aureus*) were subjected to continuous in vitro passage to generate attenuated derivatives CpS1-500 (500 passages), TpS1-260 (260 passages), and SaS9-220 (220 passages). Pathogenicity evaluation in mice and goats demonstrated that all three passaged strains exhibited significantly reduced virulence compared to their parental counterparts, with attenuated local abscess formation, pyrexia, growth retardation, and mortality. Immunization with the attenuated strains elicited robust humoral and cell-mediated immune responses, as evidenced by elevated specific antibody levels and increased IFN-γ, TNF-α, and IL-12 production. Challenge experiments revealed that CpS1-500 conferred 83.3% (5/6) protection against virulent *C. pseudotuberculosis* challenge, while TpS1-260 and SaS9-220 provided 100% (6/6) protection against their respective virulent strains. These findings demonstrate that serial subculturing is an effective approach for generating live attenuated vaccine candidates against caseous lymphadenitis-associated pathogens, with the passaged strains showing promise for further development as multivalent live vaccines for caseous lymphadenitis prevention.

## Simple Summary

Caseous lymphadenitis is a chronic bacterial disease of sheep and goats caused by three pathogens: *Corynebacterium pseudotuberculosis*, *Trueperella pyogenes*, and *Staphylococcus aureus*. It causes significant economic losses worldwide. Current vaccines offer limited protection. In this study, we weakened these three bacteria through repeated laboratory subculturing (500, 260, and 220 passages, respectively) to create live attenuated vaccine candidates. When tested in mice and goats, the attenuated strains induced strong immune responses and provided high protection against virulent challenge: 83.3% for *C. pseudotuberculosis* and 100% for both *T. pyogenes* and *S. aureus*. These results demonstrate that serial subculturing is a simple and effective method for developing multivalent live attenuated vaccines against caseous lymphadenitis, offering a practical strategy to reduce disease burden in sheep and goat production.

## 1. Introduction

CLA is a chronic infectious disease affecting sheep and goats worldwide, characterized by the formation of caseous abscesses in superficial lymph nodes and internal organs [1,2]. The disease causes significant economic losses through reduced wool production, decreased body weight, carcass condemnation, and impaired reproductive performance [3,4]. While *C. pseudotuberculosis* has long been considered the primary etiological agent of CLA [5], accumulating evidence indicates that *T. pyogenes* and *S. aureus* are also frequently isolated from CLA-like abscesses and are capable of independently inducing comparable clinical manifestations [6,7]. Our previous epidemiological investigation in Northwest China revealed that *S. aureus* (62.4%), *C. pseudotuberculosis* (17.4%), and *T. pyogenes* (15.7%) were the predominant bacteria isolated from CLA abscesses, with all three species capable of inducing caseous abscess formation through wound inoculation [8].

Beyond productivity losses, CLA-associated abscesses raise One Health concerns because all three isolates can be relevant to human health under specific exposure conditions. *C. pseudotuberculosis* is generally regarded as an infrequent but underestimated zoonotic agent; human cases present mainly as necrotizing granulomatous lymphadenitis, cutaneous or wound abscesses, and rarely pneumonia, usually following contact with ruptured abscess pus, contaminated skin wounds, raw goat milk or undercooked meat, or laboratory aerosols [9]. Occupational groups such as sheep/goat farmers, veterinarians, shearers and diagnostic staff are at greatest risk. *T. pyogenes* is not part of normal human flora and causes sporadic human infection—including wound abscesses, arthritis, sepsis and endocarditis—most often in immunocompromised patients or those with farm-animal contact, so it should be managed as an opportunistic zoonotic pathogen [10]. *S. aureus* is of particular One Health importance because commensal, methicillin-susceptible and livestock-associated MRSA clones circulate between small ruminants, dairy products, slaughterhouse environments and humans; transmission can occur through direct contact, milking, dressing of abscesses, meat handling and contaminated fomites and can be compounded by antimicrobial resistance [11]. From a preventive perspective, controlling the three pathogens at the flock level is not only a herd-health measure but also a public-health intervention: reducing abscess prevalence, diagnostic manipulation of pus, and empirical antibiotic use should lower occupational exposure and the selective pressure for resistant staphylococci. Multivalent immunization against the polymicrobial etiology of CLA therefore fits a One Health strategy linking animal health, worker safety and antimicrobial stewardship (Table 1).

Currently, no highly effective vaccine is available for CLA prevention, and existing commercial vaccines—primarily inactivated formulations targeting *C. pseudotuberculosis*—provide only partial protection [12,13]. Inactivated vaccines typically induce robust humoral immunity but are less effective at stimulating cell-mediated immune responses, which are crucial for controlling intracellular pathogens such as *C. pseudotuberculosis* [14,15]. Live attenuated vaccines, by contrast, offer the advantage of mimicking natural infection, thereby eliciting both humoral and cellular immune responses and conferring more durable protection [16,17].

Serial passage in vitro has been successfully employed to generate live attenuated vaccines for various bacterial pathogens. The most notable example is the Bacille Calmette–Guérin (BCG) vaccine, which was derived from a virulent *Mycobacterium bovis* isolate through 230 serial passages over a 13-year period (1908–1921) on glycerol-potato-bile medium [18,19]. BCG remains the only licensed vaccine against tuberculosis and has been administered to billions of individuals worldwide [20]. Similarly, the *Brucella suis* vaccine strain S2, widely used for brucellosis control in China since 1971, was obtained by serial transfer of a virulent *B. suis* biovar 1 strain [21,22]. These successful examples underscore the potential of serial passage as a practical approach for generating attenuated vaccine candidates.

For *C. pseudotuberculosis*, several attenuated mutants have been investigated as vaccine candidates. *aroQ* mutants showed reduced lymph node persistence and vaccination site reactogenicity but failed to elicit protective immunity in sheep, suggesting over-attenuation [23,24]. In contrast, the PLD-deficient mutant Toxminus, when used as a live vaccine, elicited protective immune responses associated with IFN-γ production and in vivo persistence [25,26]. For *T. pyogenes*, a live-attenuated vaccine candidate generated through sequential passage and antibiotic treatment demonstrated downregulated virulence gene expression and protective effects in mice [27]. For *S. aureus*, live-attenuated small-colony variant (SCV) vaccines have been shown to induce balanced humoral and cell-mediated responses superior to killed formulations [28,29].

Despite these advances, no live attenuated vaccine has been developed that simultaneously targets the three major CLA-associated pathogens. Given the polymicrobial etiology of CLA, a multivalent approach incorporating attenuated strains of *C. pseudotuberculosis*, *T. pyogenes*, and *S. aureus* may offer superior protection. In this study, we applied serial subculturing to attenuate clinical isolates of these three pathogens and comprehensively evaluated the virulence, immunogenicity, and protective efficacy of the resulting passaged strains in mouse and goat models, aiming to establish a foundation for developing a multivalent live vaccine against CLA.

## 2. Materials and Methods

### 2.1. Bacterial Strains and Culture Conditions

The primary strains used in this study were CpS1-1 (*C. pseudotuberculosis*), TpS1-1 (*T. pyogenes*), and SaS9-1 (*S. aureus*), previously isolated from caseous abscesses of goats with CLA and stored at −80 °C in our laboratory [8]. All strains were cultured on brain heart infusion (BHI) agar supplemented with 5% bovine serum at 36 ± 1 °C under aerobic conditions. For liquid culture, strains were grown in BHI broth supplemented with 5% bovine serum at 36 ± 1 °C with shaking at 200 rpm.

### 2.2. Serial Subculturing

Each primary strain was subjected to continuous serial passage on BHI agar supplemented with 5% bovine serum. Single colonies were picked and streaked onto fresh plates every 24 h for successive generations. The passaged derivatives obtained were CpS1-500 (500 passages), TpS1-260 (260 passages), and SaS9-220 (220 passages), selected based on preliminary pathogenicity screening in mice every 20 passages.

### 2.3. Growth Curve Determination

Overnight cultures of primary and passaged strains were inoculated at 1% (*v*/*v*) into 35 mL of BHI broth supplemented with 5% bovine serum and incubated at 36 ± 1 °C with shaking at 200 rpm. Optical density at 600 nm (OD_600_) was measured every 2 h for 24 h. Each strain was tested in triplicate, and mean values were plotted against time.

### 2.4. Animal Experiments

#### 2.4.1. Mice

Specific pathogen-free female Kunming mice (18–30 g) were purchased from Chengdu Dashuo Experimental Animal Co., Ltd., Chengdu, China. All animal procedures were approved by the Institutional Animal Care and Use Committee of Northwest A&F University.

For pathogenicity evaluation, groups of 10 mice were subcutaneously injected with 0.2 mL of bacterial suspension (10^8^ CFU/mL) of primary or passaged strains. Mice were observed for 7 days for abscess formation and mortality.

For median lethal dose (LD_50_) determination, groups of 6 mice were intraperitoneally injected with 0.2 mL of serial 10-fold dilutions of primary strains. Mortality was recorded over 14 days, and LD_50_ values were calculated using the Reed–Muench method. Passaged strains were tested at 100× LD_100_ of their respective primary strains.

#### 2.4.2. Goats

A total of 42 healthy male dairy goats (30–35 kg) seronegative for *C. pseudotuberculosis*, *T. pyogenes*, and *S. aureus* antibodies were obtained from the Experimental Animal Center of Northwest A&F University. Goats were randomly divided into 7 groups (n = 6 each): CpS1-1, CpS1-500, TpS1-1, TpS1-260, SaS9-1, SaS9-220, and PBS control. Each goat received 1 mL of bacterial suspension (10^8^ CFU/mL) or sterile PBS via subcutaneous injection in the neck.

Body temperature was recorded twice daily for 7 days post-inoculation. Body weight was measured every 7 days for 28 days. Abscess formation and mortality were recorded daily for 30 days. Maximum abscess diameters were measured for affected animals.

### 2.5. Immune Response Evaluation

Blood samples were collected from goats on days 7, 14, 21, and 28 post-inoculation. Serum was separated for specific antibody detection using ELISA kits for *C. pseudotuberculosis* (Jiangsu Jingmei Biotechnology Co., Ltd., Yancheng, China) and *S. aureus* (Shanghai Tongwei Industrial Co., Ltd., Shanghai, China), and an in-house ELISA for *T. pyogenes* as previously described [30]. Plasma was collected for cytokine analysis using commercial ELISA kits for goat IFN-γ, TNF-α, and IL-12 (Jiangsu Meimian Biotechnology Co., Ltd., Taizhou, China).

### 2.6. Challenge Protection Test

On day 30 post-immunization with passaged strains, goats were challenged subcutaneously with 1 mL of 10^8^ CFU/mL of the corresponding virulent primary strain (CpS1-1 for the CpS1-500-immunized group; TpS1-1 for the TpS1-260-immunized group; SaS9-1 for the SaS9-220-immunized group). Goats were observed for 30 days for abscess formation and mortality.

### 2.7. Statistical Analysis

Data were analyzed using SPSS 26.0 software. Differences between groups were assessed by one-way ANOVA with multiple comparisons or Student’s t*-test as appropriate. A *p*-value < 0.05 was considered statistically significant. Graphs were generated using GraphPad Prism 8.

## 3. Results

### 3.1. Selection of Attenuated Passaged Strains

Serial subculturing of the three primary strains was monitored by assessing pathogenicity in mice every 20 passages. The primary strains CpS1-1, TpS1-1, and SaS9-1 caused 100% abscess formation and 40%, 20%, and 10% mortality in mice, respectively. In contrast, the passaged derivatives caused no abscess formation or mortality at the same inoculum dose. The full dataset of pathogenicity screening across all tested passage generations is provided in Supplementary Table S1. For all three strains, abscess incidence and mortality in mice decreased progressively with increasing passage number. CpS1-500 (500 passages), TpS1-260 (260 passages), and SaS9-220 (220 passages) were identified as the derivatives with substantially reduced virulence (Table 2).

### 3.2. Growth Characteristics of Primary and Passaged Strains

Growth curve analysis revealed that all three passaged strains exhibited accelerated growth compared to their parental counterparts (Figure 1). CpS1-500 entered logarithmic phase approximately 10 h post-inoculation compared to 14 h for CpS1-1, reaching stationary phase by 16 h versus 22 h. Similarly, TpS1-260 and SaS9-220 entered logarithmic phase earlier (12 h and 6 h, respectively) than their primary strains (16 h and 8 h, respectively). These results indicate that serial passage enhanced the in vitro growth rate of all three bacterial species.

### 3.3. LD_50_ Determination in Mice

The LD_50_ values for the primary strains were determined as CpS1-1, 1.58 × 10^8^ CFU; TpS1-1, 1.93 × 10^10^ CFU; and SaS9-1, 8.97 × 10^10^ CFU (Table 3). When passaged strains were administered at 100× LD_100_ of their respective primary strains, no mortality was observed in any group over 14 days, confirming the substantial reduction in lethal virulence.

### 3.4. Pathogenicity of Primary and Passaged Strains in Goats

#### 3.4.1. Body Temperature

Primary strains CpS1-1, TpS1-1, and SaS9-1 induced significant pyrexia (>39.5 °C) lasting 6, 4, and 2 days, respectively. In contrast, passaged strains CpS1-500, TpS1-260, and SaS9-220 caused minimal temperature elevation, with only CpS1-500 inducing a transient fever (>40 °C) on day 1 that returned to normal by day 2 (Figure 2).

#### 3.4.2. Body Weight

Over the 28-day observation period, goats inoculated with primary strains showed significantly reduced average daily weight gain compared to PBS controls. Goats receiving passaged strains exhibited initial growth retardation during the first 14 days but subsequently recovered to weight gain levels comparable to controls, indicating that attenuation reduced the negative impact on growth performance (Figure 3).

#### 3.4.3. Abscess Formation and Mortality

In all groups, abscesses were exclusively localized at the injection site in the neck region. No additional superficial abscesses were observed at other anatomical sites throughout the 30-day observation period. Each affected goat developed a single abscess at the injection site. Representative photographs of abscesses at the injection site in goats inoculated with the strains are shown in Figures S1–S6 (Supplementary Materials). Primary strains CpS1-1, TpS1-1, and SaS9-1 caused 100% (6/6) abscess formation in inoculated goats, with mortality rates of 33.3% (2/6), 16.7% (1/6), and 0%, respectively. In contrast, CpS1-500 caused only 16.7% (1/6) abscess formation with no mortality, while TpS1-260 and SaS9-220 caused no abscess formation or mortality (Figure 4A). Maximum abscess diameters were significantly smaller in the CpS1-500 group compared to the CpS1-1 group (*p* < 0.01) (Figure 4B). For goats inoculated with the primary strains, the mean maximum abscess diameters were 6.71 ± 3.02 cm (range: 2.15–10.57 cm) in the CpS1-1 group, 5.79 ± 0.94 cm (range: 3.22–7.16 cm) in the TpS1-1 group, and 5.09 ± 0.68 cm (range: 4.33–5.96 cm) in the SaS9-1 group (Table 4). In the CpS1-500 group, only one goat developed an abscess at the injection site, with a maximum diameter of 1.12 cm. No abscesses developed at the injection site or elsewhere in goats inoculated with TpS1-260 or SaS9-220.

**Table 4:** Clinical outcomes in goats after subcutaneous inoculation with primary or passaged strains: abscess development, mortality, and maximum abscess diameter. * Values marked with asterisks indicate abscess diameter at time of death.

Strain	Goats (n)	Abscess Cases	Deaths	Maximum Abscess Diameter (cm)
CpS1-1	6	6	2	7.41, 9.56, 2.15, 3.68, 10.57, 6.88
CpS1-500	6	1	0	1.12, 0, 0, 0, 0, 0
TpS1-1	6	6	1	6.58, 5.67, 3.22 *, 7.16, 6.08, 6.05
TpS1-260	6	0	0	0, 0, 0, 0, 0, 0
SaS9-1	6	6	0	5.44, 4.38, 5.17, 5.96, 4.33, 5.26
SaS9-220	6	0	0	0, 0, 0, 0, 0, 0

**Figure 4:**
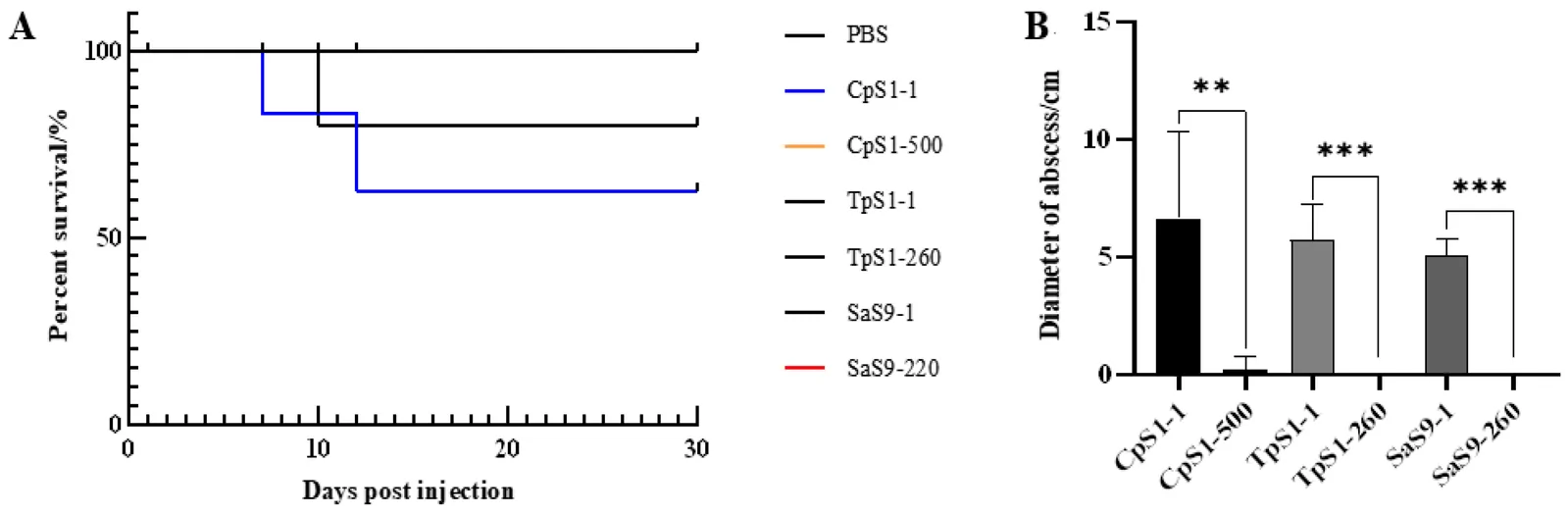
Survival curve (**A**) and comparison of abscess diameter (**B**) in experimental goats. **: *p* < 0.01; ***: *p* < 0.001.

### 3.5. Immune Responses to Primary and Passaged Strains in Goats

#### 3.5.1. Humoral Immune Responses

All three passaged strains induced significant specific antibody responses comparable to their primary counterparts (Figure 5A, Figure 6A and Figure 7A). For C. pseudotuberculosis, both CpS1-1 and CpS1-500 groups showed significantly elevated antibody levels compared to controls at all time points (*p* < 0.001). Interestingly, at day 14, the CpS1-500 group exhibited significantly higher antibody levels than the CpS1-1 group, though this trend reversed by day 28. For *T. pyogenes* and *S. aureus*, no significant differences in antibody levels were observed between primary and passaged strain groups at most time points (*p* > 0.05), indicating that serial passage did not compromise the immunogenicity of these strains.

#### 3.5.2. Cell-Mediated Immune Responses

IFN-γ levels were significantly elevated in all primary and passaged strain groups compared to controls (*p* < 0.001) (Figure 5B, Figure 6B and Figure 7B). No significant differences in IFN-γ production were observed between primary and passaged strain groups for C. pseudotuberculosis (except day 21) and *T. pyogenes*, indicating that the ability to induce Th1-type responses was preserved after attenuation.

TNF-α production showed distinct patterns among the three pathogens (Figure 5C, Figure 6C and Figure 7C). For CpS1-500, TNF-α levels were significantly lower than CpS1-1 at days 14 and 28 (*p* < 0.001), suggesting reduced inflammatory tissue damage. For TpS1-260, TNF-α levels were comparable to TpS1-1 at early time points but returned to control levels by day 28. For SaS9-220, TNF-α levels remained at control levels throughout the observation period, significantly lower than SaS9-1.

IL-12 production was significantly elevated in all immunized groups compared to controls, with no significant differences between primary and passaged strain groups for any of the three pathogens (Figure 5D, Figure 6D and Figure 7D), indicating that the intracellular lifestyle and ability to stimulate Th1 differentiation were maintained after attenuation.

### 3.6. Protective Efficacy of Passaged Strains

Challenge experiments demonstrated that immunization with passaged strains conferred significant protection against virulent challenge (Table 5). CpS1-500-immunized goats showed 83.3% (5/6) protection against CpS1-1 challenge, with only one goat developing an abscess (maximum diameter 2.4 cm) that matured and ruptured on day 6 post-challenge. TpS1-260 and SaS9-220 provided complete protection (100%, 6/6) against their respective virulent strains, with no abscess formation or mortality observed.

## 4. Discussion

Serial passage in vitro has long been recognized as a practical approach for generating live attenuated vaccines, with BCG representing the most successful and enduring example [18,19,31]. The BCG vaccine was derived through 230 serial passages of a virulent *M. bovis* isolate over 13 years, resulting in cumulative mutations that progressively reduced virulence while preserving immunogenicity [32,33]. Similarly, the *Brucella suis* vaccine strain S2 was obtained by serial transfer and has been used extensively in China since 1971 [21,22]. In the present study, we applied this classical approach to generate attenuated derivatives of the three major CLA-associated pathogens—*C. pseudotuberculosis*, *T. pyogenes*, and *S. aureus*—and comprehensively evaluated their potential as live vaccine candidates.

The attenuation achieved through serial passage was substantial for all three species. CpS1-500, TpS1-260, and SaS9-220 all exhibited markedly reduced pathogenicity in both mice and goats compared to their parental strains. Notably, the number of passages required for effective attenuation differed among the three species: 500 passages for *C. pseudotuberculosis* versus 260 and 220 passages for *T. pyogenes* and *S. aureus*, respectively. This differential rate of attenuation likely reflects species-specific differences in genomic plasticity, mutation rates, and the genetic basis of virulence. *C. pseudotuberculosis*, being a facultative intracellular pathogen with multiple virulence determinants including PLD, mycolic acid, and iron-regulated factors [34,35], may require more extensive genetic changes to achieve comparable attenuation. This observation is consistent with the finding that *aroQ* mutants of *C. pseudotuberculosis* may be overly attenuated for use as vaccines [23,24], suggesting a delicate balance between sufficient attenuation and retained immunogenicity for this pathogen.

At the molecular level, the differential attenuation thresholds likely reflect the number and functional redundancy of virulence determinants carried by each species. In C. pseudotuberculosis, phospholipase D (PLD), mycolic acids, and iron-regulated factors act in concert to promote intracellular survival and dissemination; serial passage on artificial media, which lacks host-derived selective pressures, may favor the stepwise silencing of these determinants [34,35]. In *T. pyogenes*, pyolysin (PLO) is the dominant cholesterol-dependent cytolysin responsible for tissue necrosis and abscess formation; the complete loss of abscess-inducing ability in TpS1-260 suggests that PLO expression or secretion was functionally abrogated during passage [36]. In S. aureus, the accessory gene regulator (*agr*) quorum-sensing system controls the expression of α-hemolysin, leukocidins, and other exotoxins; repeated subculture in nutrient-rich medium is known to select for agr-defective variants with markedly reduced toxin production and virulence, which is consistent with the attenuated phenotype of SaS9-220 [37,38].

The growth curve analysis revealed that all three passaged strains exhibited accelerated in vitro growth compared to their parental counterparts. This phenomenon has been observed in other serially passaged pathogens and may reflect adaptation to laboratory culture conditions, including loss of virulence-associated genes that are costly to maintain [37]. The enhanced growth rate may also contribute to the safety profile of these strains, as rapid in vitro growth does not necessarily correlate with in vivo fitness [38].

The immune response analysis demonstrated that all three passaged strains retained robust immunogenicity despite attenuation. Both humoral and cell-mediated immune responses were induced at levels comparable to or only slightly reduced from the primary strains. This is particularly important for *C. pseudotuberculosis*, where cell-mediated immunity, particularly IFN-γ production, plays a critical role in protection [14,15,39]. Our finding that CpS1-500 induced IFN-γ levels comparable to CpS1-1 suggests that the attenuated strain retains the ability to stimulate Th1-type responses, which is essential for controlling intracellular infection [40,41]. This is consistent with previous reports that live attenuated *C. pseudotuberculosis* strains elicit protective immune responses associated with IFN-γ production [25,26].

The differential TNF-α responses between primary and passaged strains are noteworthy. TNF-α is a pro-inflammatory cytokine involved in granuloma formation and tissue necrosis [42]. The significantly lower TNF-α levels observed in goats receiving passaged strains, particularly CpS1-500 and SaS9-220, suggest reduced inflammatory tissue damage, which may contribute to the improved safety profile of these vaccine candidates. This is consistent with the reduced abscess sizes and absence of mortality observed in these groups.

IL-12, a key cytokine for Th1 differentiation and control of intracellular pathogens [43,44], was induced at comparable levels by both primary and passaged strains across all three species. This indicates that the attenuated strains maintain the ability to stimulate protective cell-mediated immune responses, which is crucial for vaccine efficacy against facultative intracellular pathogens like *C. pseudotuberculosis*.

The challenge protection results are particularly encouraging. CpS1-500 provided 83.3% protection against virulent *C. pseudotuberculosis* challenge, while TpS1-260 and SaS9-220 provided complete protection (100%). These protection rates compare favorably with those reported for other live attenuated *C. pseudotuberculosis* vaccines. For instance, Hodgson et al. reported that the PLD-negative Toxminus strain, when used as a live vaccine, elicited protective immunity in sheep [25,26]. Simmons et al. found that *aroQ* mutants failed to protect sheep from infection but did reduce clinical severity [23,24]. The superior protection conferred by TpS1-260 and SaS9-220 may reflect the predominantly extracellular lifestyle of these pathogens, against which antibody-mediated immunity may be more effective [36,45].

The successful attenuation of all three pathogens through serial passage, combined with their retained immunogenicity and protective efficacy, suggests that a multivalent live vaccine incorporating CpS1-500, TpS1-260, and SaS9-220 could be developed for comprehensive CLA prevention. Such a vaccine would address the polymicrobial etiology of CLA [6,7,8] and potentially offer superior protection compared to monovalent vaccines targeting only *C. pseudotuberculosis*. The safety profile of the passaged strains—minimal pyrexia, reduced growth retardation, and absence of mortality—supports their potential for field application.

Several limitations of this study should be acknowledged. First, the genetic basis of attenuation in the passaged strains remains to be elucidated. Whole-genome sequencing and comparative genomic analysis would help identify the specific mutations responsible for reduced virulence and may inform future rational vaccine design [46]. Second, the duration of protective immunity and the optimal immunization schedule require further investigation. The efficacy of combining the three attenuated strains into a single multivalent formulation needs to be evaluated, as potential interference between vaccine components could occur. Third, challenge experiments in this study used only homologous parental strains; cross-protection against heterologous isolates from different geographical regions or different host species remains to be determined. Fourth, all animal experiments were conducted only in goats, which represent a single host system. Given that ovine caseous lymphadenitis differs from the caprine form in lesion distribution, immune response dynamics, and production-system context, the safety, immunogenicity, and protective efficacy of the passaged strains must be further validated in sheep under field conditions before any practical application. Finally, in sheep and goats, CLA can manifest as a visceral form characterized by abscesses in the lungs, liver, kidneys, spleen, and thoracic or abdominal lymph nodes, in addition to the more readily detectable superficial form [47,48,49]. Internal abscesses are typically not visible externally and can only be definitively detected through post-mortem examination [49,50,51]. In the present study, the reasons for observing animals for 30 days post-inoculation without performing necropsy were (1) the experimental design required prolonged observation of the animals to monitor whether delayed abscess formation would occur at the injection site; (2) it was necessary to evaluate the duration of protective immunity and to monitor antibody responses over time, which required the animals to remain alive throughout the study; and (3) during the entire experimental period, all animals were monitored daily for clinical signs, including rectal temperature and general condition, and no abnormalities suggestive of systemic infection or visceral involvement were observed. However, this approach precluded the detection of potential visceral lesions. Given that the internal form of CLA is more common in sheep and that visceral involvement is a major contributor to mortality and carcass condemnation [47,48], future studies should incorporate necropsy at defined endpoints to comprehensively evaluate the protective efficacy of the attenuated vaccine candidates against both superficial and visceral CLA. This would also enable histopathological confirmation of the nature and severity of any internal lesions, as has been performed in previous CLA pathogenesis studies [52].

In conclusion, this study demonstrates that serial subculturing is an effective approach for generating live attenuated vaccine candidates against the three major CLA-associated pathogens. The passaged strains CpS1-500, TpS1-260, and SaS9-220 exhibit substantially reduced virulence while retaining robust immunogenicity and conferring significant protection against virulent challenge. These findings provide a foundation for the development of a multivalent live vaccine for the prevention of CLA, addressing a critical gap in the control of this economically important disease.

## 5. Conclusions

In conclusion, this study demonstrates that continuous serial subculturing is an effective approach for generating live attenuated vaccine candidates against the three major CLA-associated pathogens. The passaged strains—CpS1-500, TpS1-260, and SaS9-220—exhibited substantially reduced pathogenicity in both mice and goats while retaining robust immunogenicity, inducing both humoral and cell-mediated immune responses comparable to those elicited by their parental strains. Notably, immunization with CpS1-500, TpS1-260, and SaS9-220 conferred 83.3%, 100%, and 100% protection, respectively, against virulent challenge in goats, highlighting their strong potential as safe and effective live vaccine candidates for the prevention of ovine caseous lymphadenitis.

## Figures and Tables

**Figure 1:**
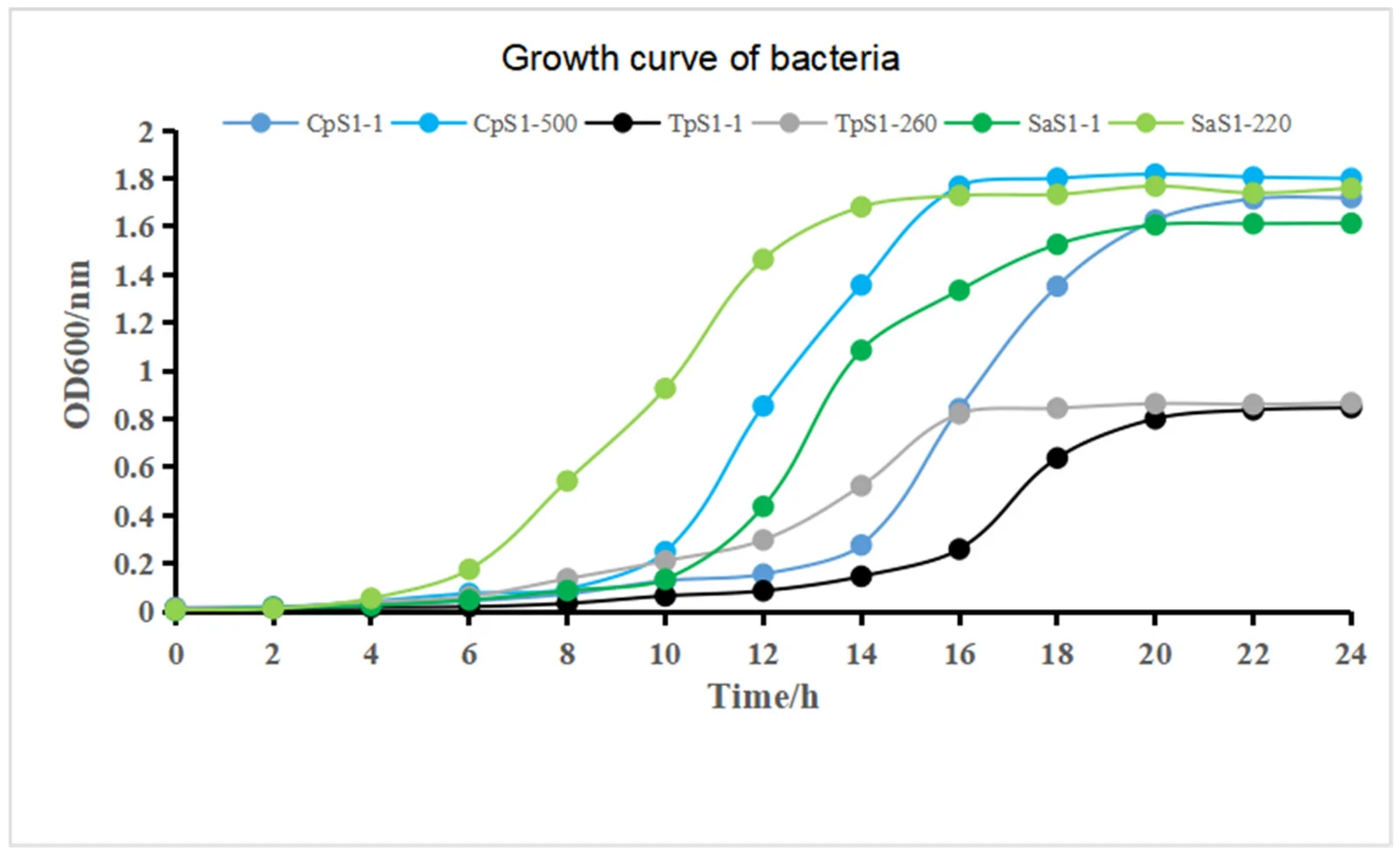
Growth curves of primary and serially passaged strains in BHI broth supplemented with 5% bovine serum. Optical density at 600 nm was recorded every 2 h for 24 h.

**Figure 2:**
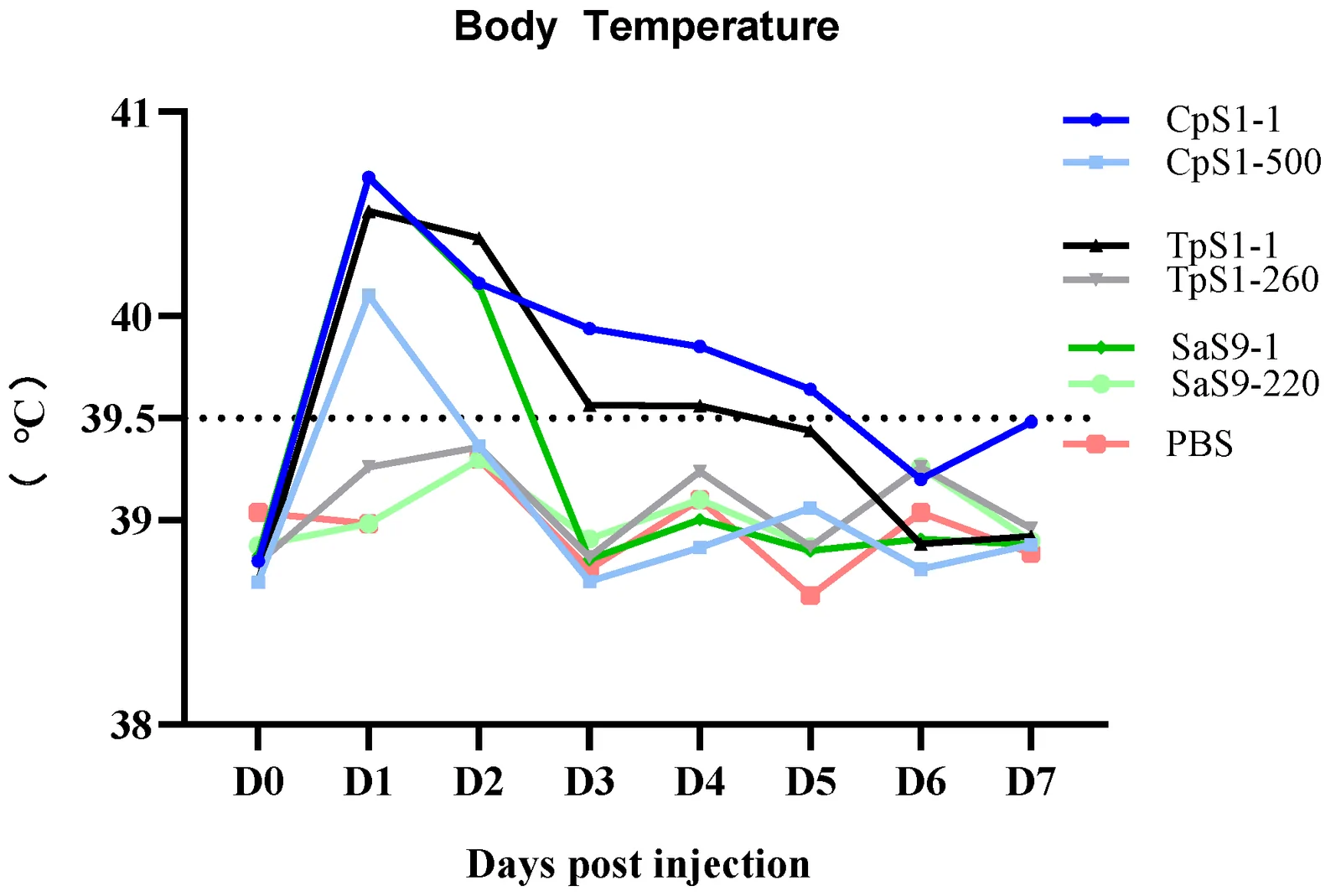
Rectal temperature changes in goats during the first 7 days after subcutaneous inoculation with primary or passaged strains. Data are shown as mean ± SD.

**Figure 3:**
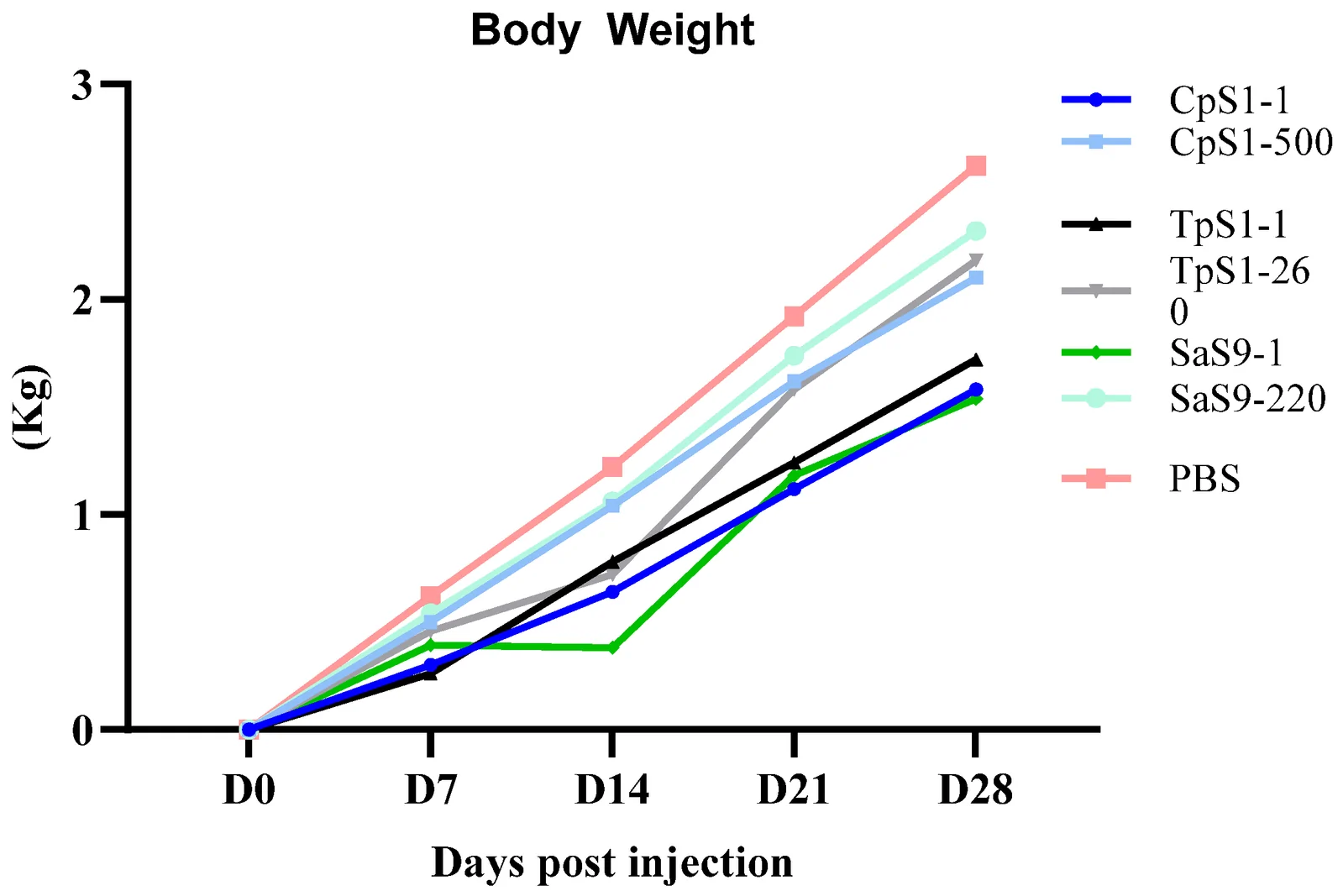
Body weight changes in goats over a 28-day period after inoculation with primary or passaged strains.

**Figure 5:**
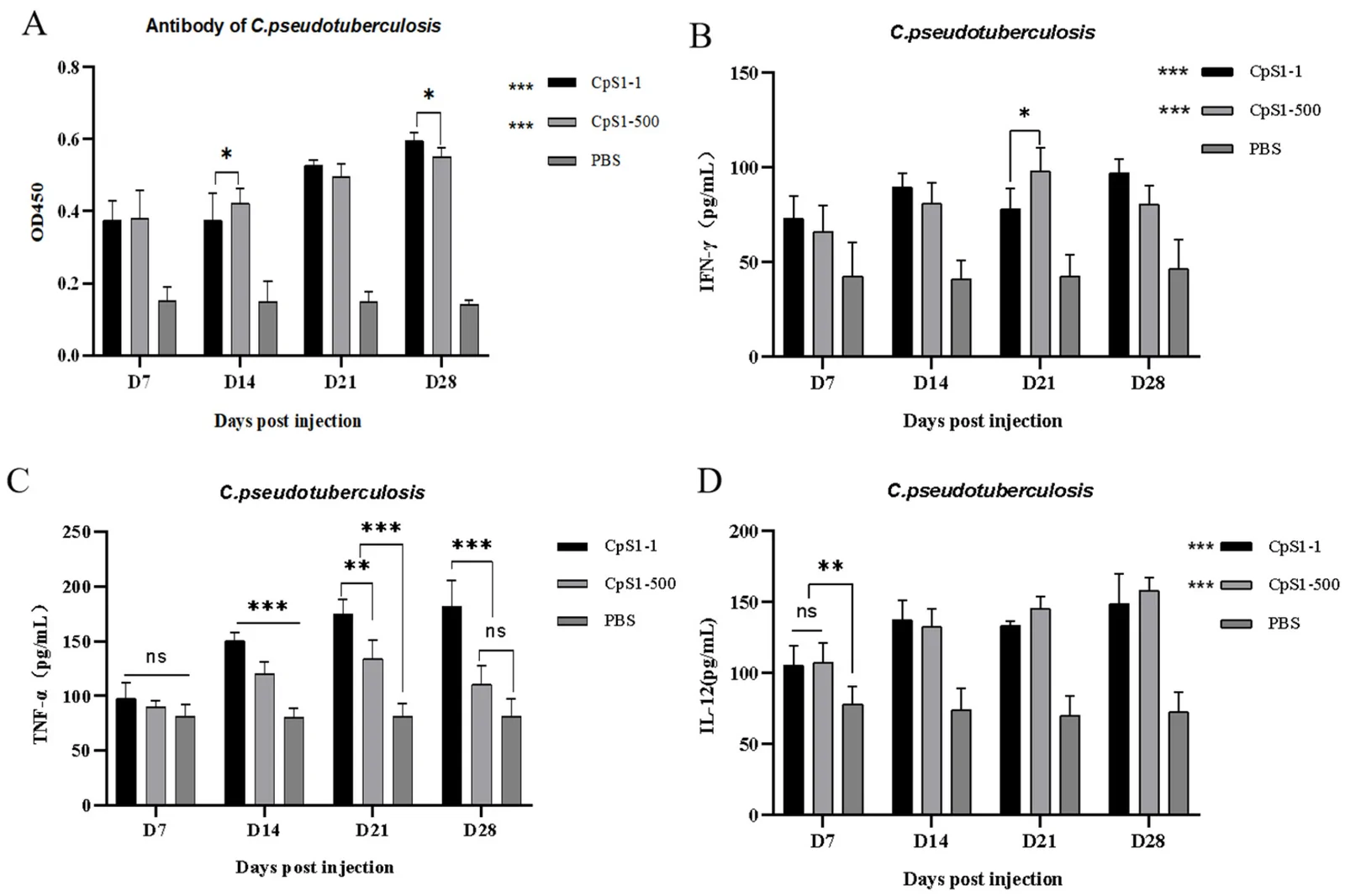
Immune responses of experimental goats to different strains of *C. pseudotuberculosis.* Antibody (**A**); IFN-γ (**B**); TNF-α (**C**); IL-12 (**D**); ns: *p* > 0.05; *: *p* < 0.05; **: *p* < 0.01; ***: *p* < 0.001.

**Figure 6:**
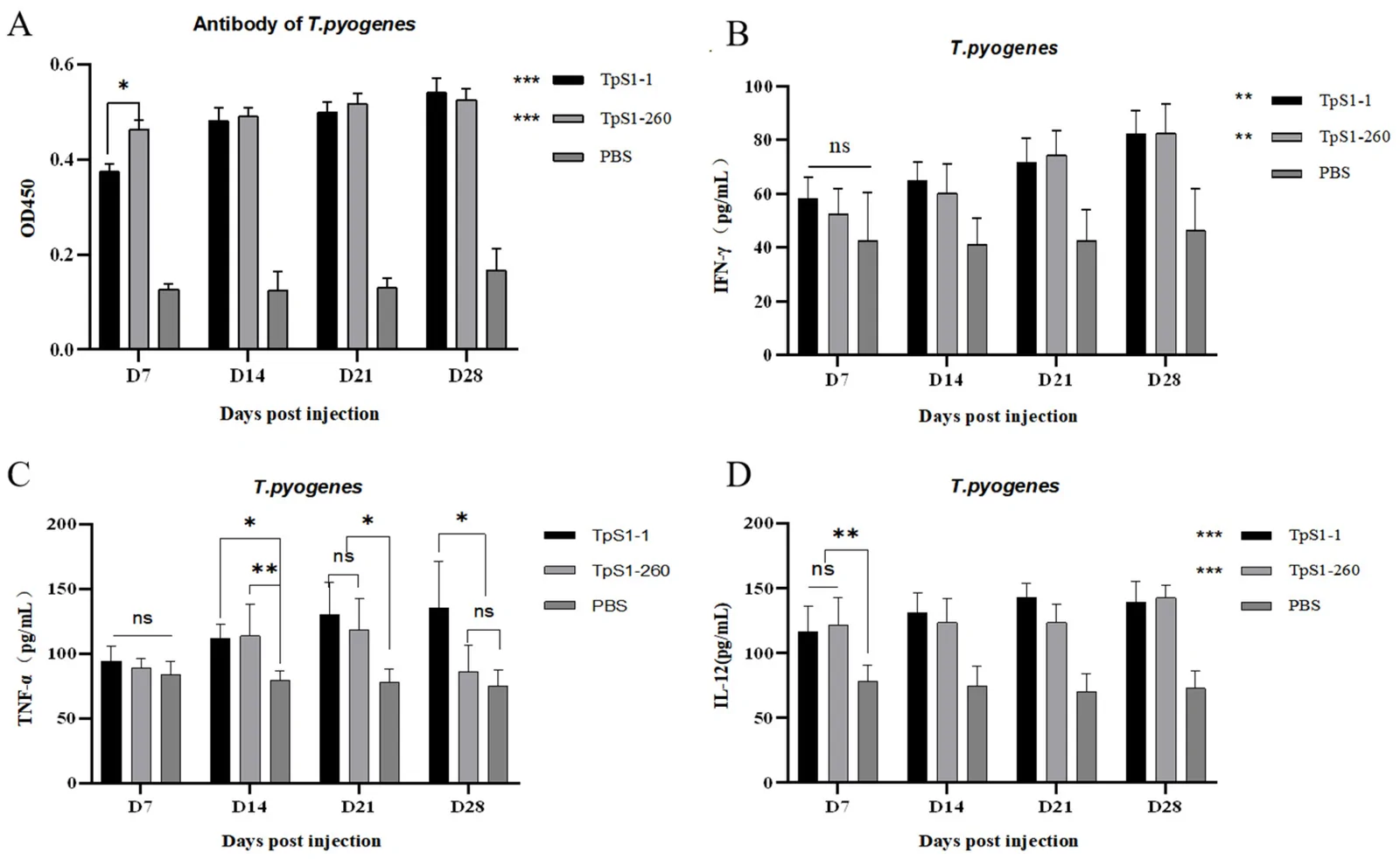
Immune responses of experimental goats to different strains of *T. pyogenes.* Antibody (**A**); IFN-γ (**B**); TNF-α (**C**); IL-12 (**D**); ns: *p* > 0.05; *: *p* < 0.05; **: *p* < 0.01; ***: *p* < 0.001.

**Figure 7:**
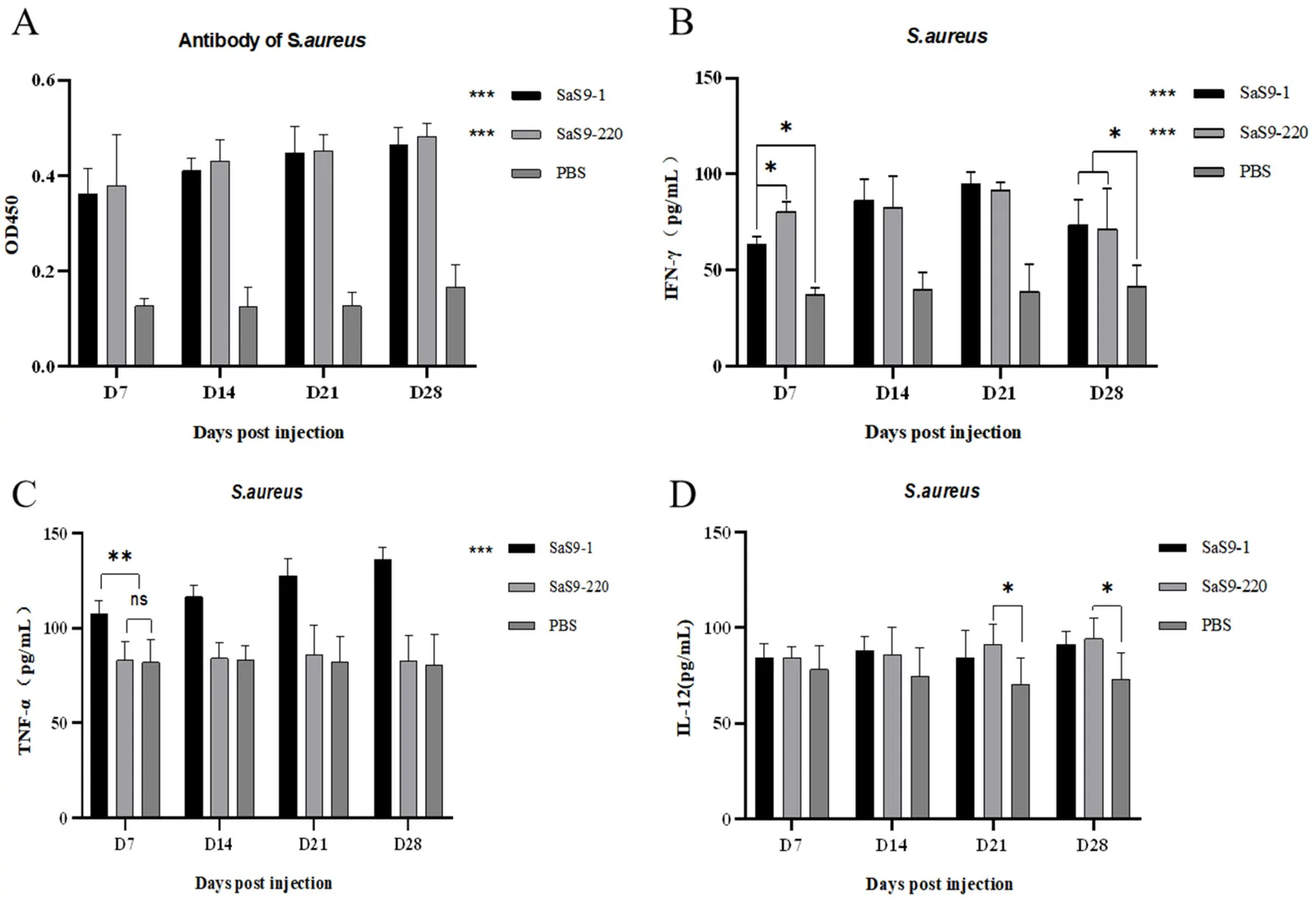
Immune responses of experimental goats to different strains of *S. aureus.* Antibody (**A**); IFN-γ (**B**); TNF-α (**C**); IL-12 (**D**); ns: *p* > 0.05; *: *p* < 0.05; **: *p* < 0.01; ***: *p* < 0.001.

**Table 1:** Overview of the three major caseous lymphadenitis (CLA)-associated microorganisms.

Microorganism	Primary Natural Host(s)	Relevance to CLA
*C. pseudotuberculosis*	Sheep, goats (small ruminants); occasionally horses, cattle, and camelids [9]	Classic pathogen of CLA, facultative intracellular bacterium
*T. pyogenes*	Cattle, sheep, goats, pigs and horses [10]	Common isolate from purulent lesions of CLA, producing pyolysin
*S. aureus*	Small ruminants (sheep, goats); mammals including humans are widespread [11]	The predominant bacteria in this epidemiological study

**Table 2:** In vivo pathogenicity of primary and serially passaged strains in mice: abscess incidence and mortality after subcutaneous inoculation.

Strain	Mice (n)	Abscess Incidence (%)	Mortality (%)
CpS1-1	10	100 (10/10)	40 (4/10)
CpS1-500	10	0	0
TpS1-1	10	100 (10/10)	20 (2/10)
TpS1-260	10	0	0
SaS9-1	10	100 (10/10)	10 (1/10)
SaS9-220	10	0	0

**Table 3:** Determination of the median lethal dose (LD50) of primary strains in mice after intraperitoneal injection.

Strain	Concentration (CFU/mL)	Deaths (n = 6)
CpS1-1	5.02 × 10^10^	6
	3.49 × 10^9^	5
	2.39 × 10^8^	3
	1.64 × 10^7^	2
	1.12 × 10^6^	0
LD_50_	1.58 × 10^8^ CFU	
TpS1-1	8.10 × 10^13^	6
	1.25 × 10^12^	4
	1.96 × 10^10^	3
	3.06 × 10^8^	2
	4.78 × 10^6^	0
LD_50_	1.93 × 10^10^ CFU	
SaS9-1	6.67 × 10^12^	6
	5.05 × 10^11^	4
	3.83 × 10^10^	2
	2.90 × 10^9^	1
	2.19 × 10^8^	0
LD_50_	8.97 × 10^10^ CFU	

**Table 5:** Protective efficacy of immunization with passaged strains against homologous virulent challenge in goats: morbidity, mortality, and protection rate.

Immunization Strain	Challenge Strain	Goats (n)	Morbidity (%)	Mortality (%)	Protection (%)
CpS1-500	CpS1-1	6	16.7 (1/6)	0	83.3 (5/6)
TpS1-260	TpS1-1	6	0	0	100 (6/6)
SaS9-220	SaS9-1	6	0	0	100 (6/6)

## Data Availability

The original contributions presented in this study are included in the article/Supplementary Material. Further inquiries can be directed to the corresponding authors.

## Supplementary Materials

The following supporting information can be downloaded at: https://www.mdpi.com/article/10.3390/vetsci13100996/s1, Figure S1: Representative photographs of abscesses at the injection site in goats inoculated with the CpS1-1; Figure S2: Representative photographs of abscesses at the injection site in goats inoculated with the CpS1-500; Figure S3: Representative photographs of abscesses at the injection site in goats inoculated with the TpS1-1; Figure S4: Representative photographs of abscesses at the injection site in goats inoculated with the TpS1-260; Figure S5: Representative photographs of abscesses at the injection site in goats inoculated with the SaS9-1; Figure S6: Representative photographs of abscesses at the injection site in goats inoculated with the SaS9-220; Table S1: Pathogenicity test results of different passage strains in mice.
