# Supplementary material for: Development and Evaluation of Artificially Attenuated Corynebacterium pseudotuberculosis, Trueperella pyogenes, and Staphylococcus aureus Strains as Live Vaccine Candidates Against Ovine Caseous Lymphadenitis
Source: Vet Sci. 2026 Sep 22;13(10):996. doi: 10.3390/vetsci13100996 (PMC13709487; doi:10.3390/vetsci13100996)
Supplement: Supplementary file 1 [file vetsci-13-00996-s001.zip › vetsci-4560453-supplementary.pdf]

Table S1 Pathogenicity test results of different passage strains in mice (n = 10)

Strain	Passage	Number of affected animals	Morbidity rate (%)	Number of deaths	Mortality rate (%)
CpS1	CpS1-1	10	100	4	40
	CpS1-20	10	100	2	20
	CpS1-40	10	100	2	20
	CpS1-60	9	90	0	0
	CpS1-80	9	90	0	0
	CpS1-100	8	80	0	0
	CpS1-120	8	80	0	0
	CpS1-140	8	80	0	0
	CpS1-160	7	70	0	0
	CpS1-180	6	60	0	0
	CpS1-200	6	60	0	0
	CpS1-220	6	60	0	0
	CpS1-240	6	60	0	0
	CpS1-260	4	40	0	0
	CpS1-280	5	50	0	0
	CpS1-300	4	40	0	0
	CpS1-320	2	20	0	0
	CpS1-340	1	10	0	0
	CpS1-360	1	10	0	0
	CpS1-380	2	20	0	0
	CpS1-400	1	10	0	0
	CpS1-420	0	0	0	0
	CpS1-440	0	0	0	0
	CpS1-460	0	0	0	0
	CpS1-480	0	0	0	0
	CpS1-500	0	0	0	0
TpS1	TpS1-1	10	100	2	20
	TpS1-20	10	100	2	20
	TpS1-40	8	80	1	10
	TpS1-60	7	70	0	0
	TpS1-80	8	80	1	10
	TpS1-100	5	50	0	0
	TpS1-120	3	30	0	0
	TpS1-140	2	20	0	0
	TpS1-160	1	10	0	0
	TpS1-180	0	0	0	0
	TpS1-200	0	0	0	0
	TpS1-220	0	0	0	0

	TpS1-240	0	0	0	0
	TpS1-260	0	0	0	0
SaS9	SaS9-1	10	100	1	10
	SaS9-20	9	90	1	0
	SaS9-40	7	70	0	0
	SaS9-60	3	30	0	0
	SaS9-80	2	20	0	0
	SaS9-100	1	10	0	0
	SaS9-120	1	10	0	0
	SaS9-140	0	0	0	0
	SaS9-160	0	0	0	0
	SaS9-180	0	0	0	0
	SaS9-200	0	0	0	0
	SaS9-220	0	0	0	0

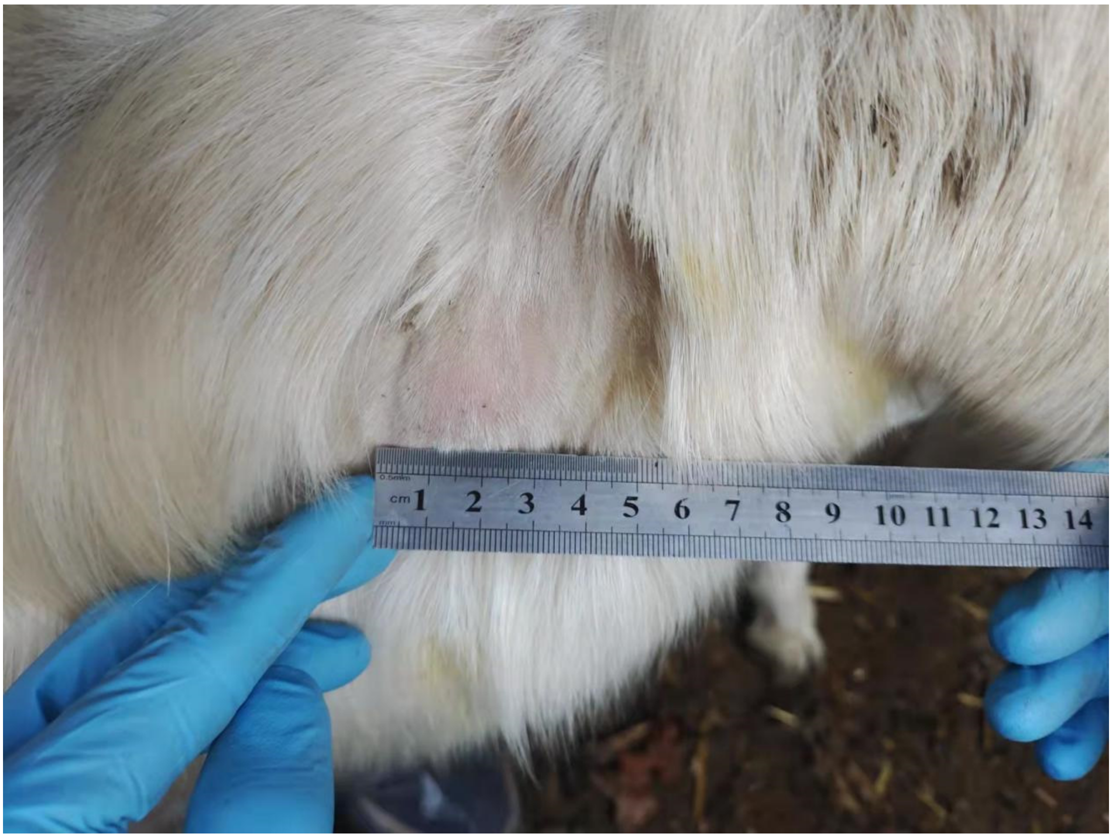

Figure S1 Representative photographs of abscesses at the injection site in goats inoculated with the CpS1-1

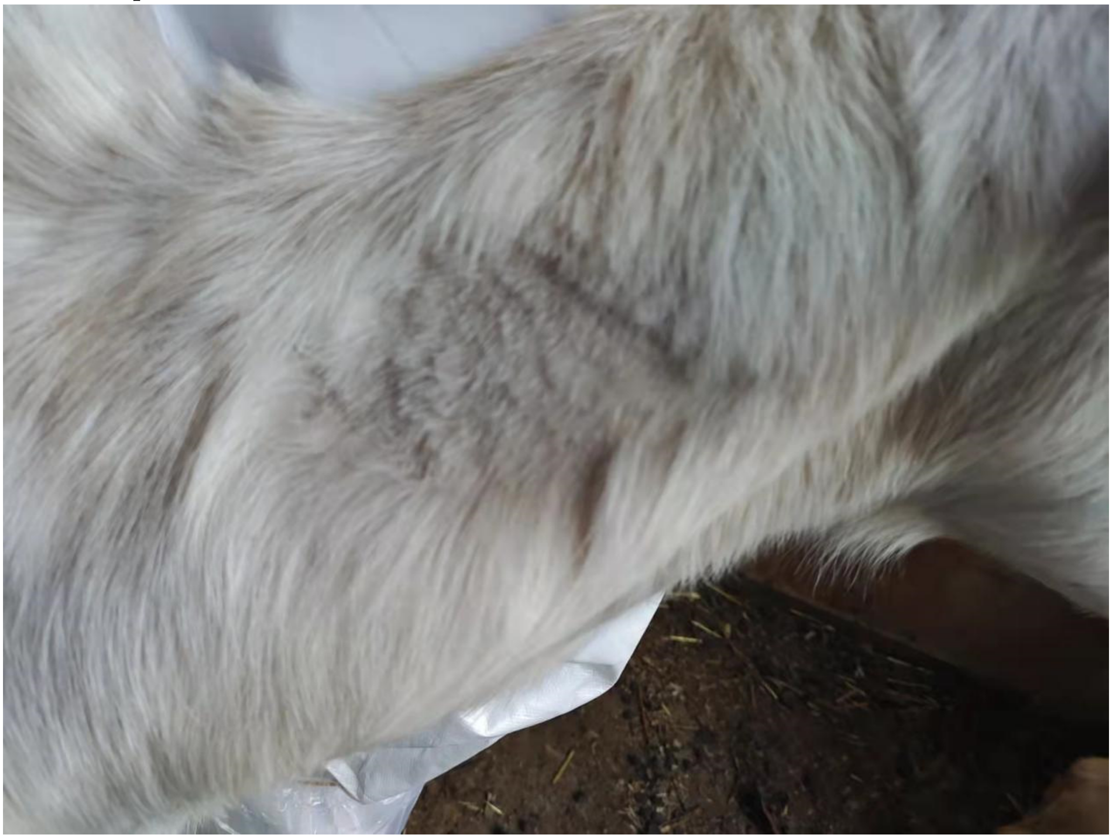

Figure S2 Representative photographs of abscesses at the injection site in goats inoculated with the CpS1-500

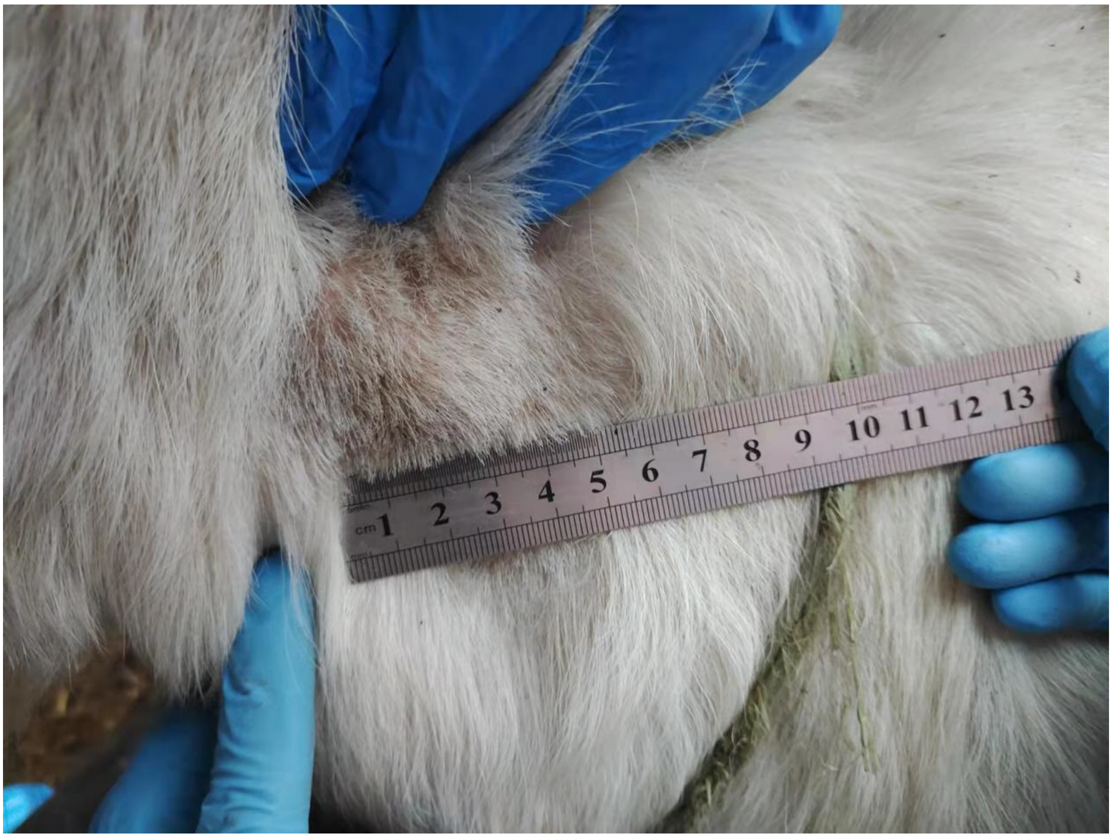

Figure S3 Representative photographs of abscesses at the injection site in goats inoculated with the TpS1-1

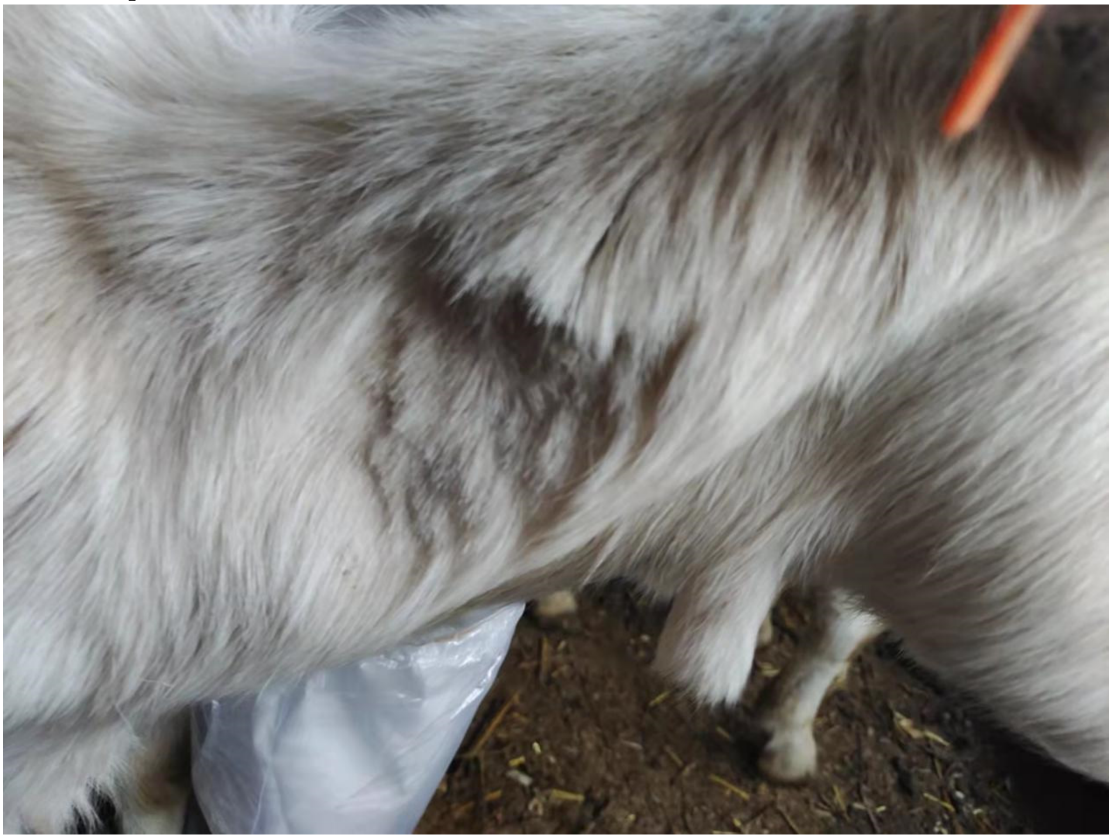
Figure S4 Representative photographs of abscesses at the injection site in goats inoculated with the TpS1-260

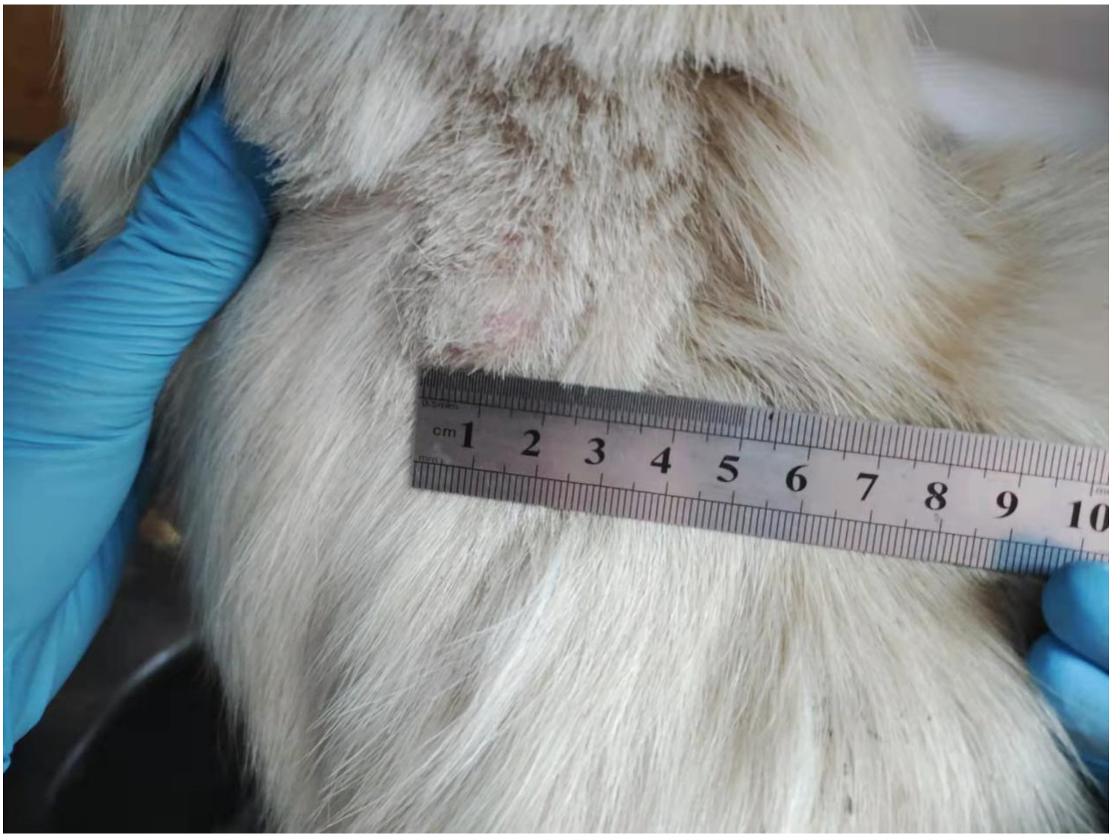

Figure S5 Representative photographs of abscesses at the injection site in goats inoculated with the SaS9-1

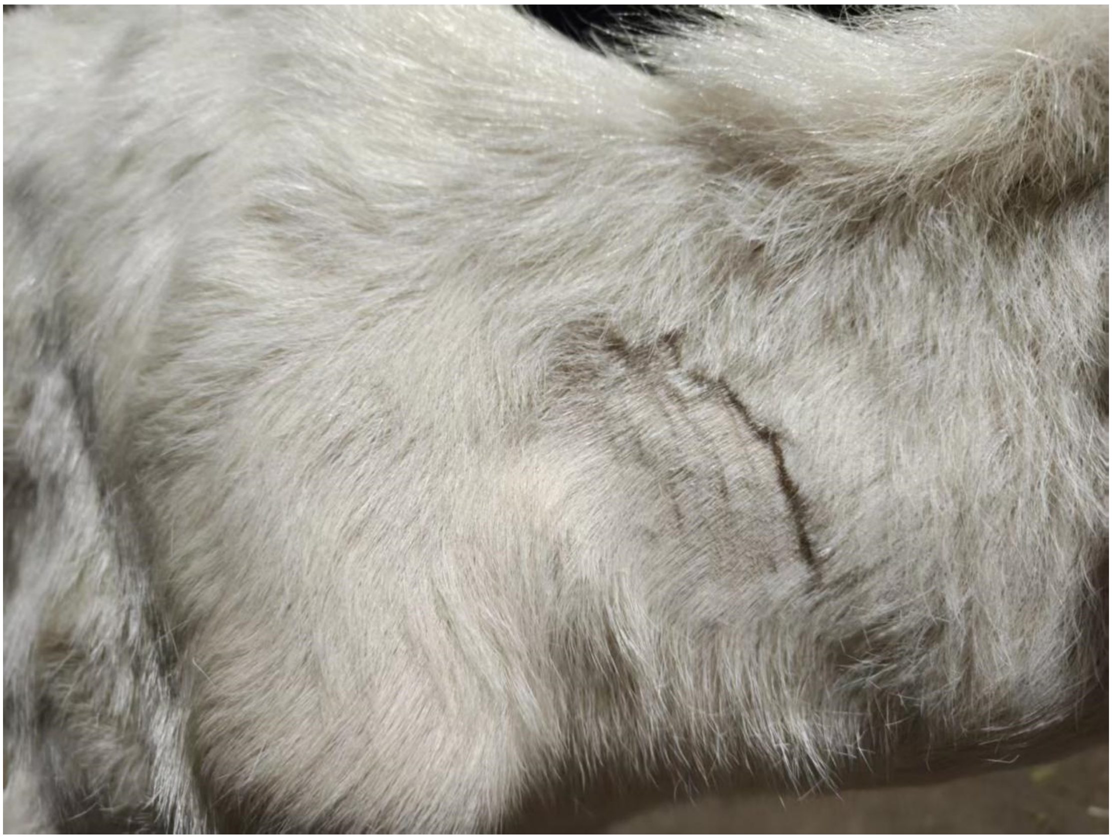

Figure S6 Representative photographs of abscesses at the injection site in goats inoculated with the SaS9-220
